# Metachronous colorectal cancer following segmental or extended colectomy in Lynch syndrome: a systematic review and meta-analysis

**DOI:** 10.1007/s10689-017-0062-2

**Published:** 2017-11-30

**Authors:** Salim S. Malik, Mark P. Lythgoe, Mark McPhail, Kevin J. Monahan

**Affiliations:** 1 Imperial College London, London, UK; 2 Family History of Bowel Cancer Clinic, West Middlesex University Hospital, Chelsea and Westminster Hospitals NHS Trust, London, TW7 6AF UK

**Keywords:** Lynch syndrome, Metachronous colorectal cancer, Amsterdam criteria, Colorectal cancer, Colectomy

## Abstract

Around 5% of colorectal cancers are due to mutations within DNA mismatch repair genes, resulting in Lynch syndrome (LS). These mutations have a high penetrance with early onset of colorectal cancer at a mean age of 45 years. The mainstay of surgical management is either a segmental or extensive colectomy. Currently there is no unified agreement as to which management strategy is superior due to limited conclusive empirical evidence available. A systematic review and meta- analysis to evaluate the risk of metachronous colorectal cancer (MCC) and mortality in LS following segmental and extensive colectomy. A systematic review of the PubMed database was conducted. Studies were included/ excluded based on pre-specified criteria. To assess the risk of MCC and mortality attributed to segmental or extensive colectomies, relative risks (RR) were calculated and corresponding 95% confidence intervals (CI). Publication bias was investigated using funnel plots. Data about mortality, as well as patient ascertainment [Amsterdam criteria (AC), germline mutation (GM)] were also extracted. Statistical analysis was conducted using the R program (version 3.2.3). The literature search identified 85 studies. After further analysis ten studies were eligible for inclusion in data synthesis. Pooled data identified 1389 patients followed up for a mean of 100.7 months with a mean age of onset of 45.5 years of age. A total 1119 patients underwent segmental colectomies with an absolute risk of MCC in this group of 22.4% at the end of follow-up. The 270 patients who had extensive colectomies had a MCC absolute risk of 4.7**%** (0% in those with a panproctocolecomy). Segmental colectomy was significantly associated with an increased relative risk of MCC (RR = 5.12; 95% CI 2.88–9.11; Fig. 1), although no significant association with mortality was identified (RR = 1.65; 95% CI 0.90–3.02). There was no statistically significant difference in the risk of MCC between AC and GM cohorts (p = 0.5, Chi-squared test). In LS, segmental colectomy results in a significant increased risk of developing MCC. Despite the choice of segmental or extensive colectomies having no statistically significant impact on mortality, the choice of initial surgical management can impact a patient’s requirement for further surgery. An extensive colectomy can result in decreased need for further surgery; reduced hospital stays and associated costs. The significant difference in the risk of MCC, following segmental or extensive colectomies should be discussed with patients when deciding appropriate management. An individualised approach should be utilised, taking into account the patient’s age, co-morbidities and genotype. In order to determine likely germline-specific effects, or a difference in survival, larger and more comprehensive studies are required.

## Introduction

In 2012, 694,000 deaths worldwide were attributed to colorectal cancer (CRC) alone [1]. Lynch syndrome (LS), the most common form of hereditary CRC, causes 3.1% of all CRC, and is associated with a high rate of either metachronous (MCC) or synchronous CRC [2].

LS is an autosomal dominantly inherited condition which occurs due to a germline mutation in one or more DNA mismatch repair genes (MMR) (*MLH1, MSH2, MSH6* or *PMS2* genes) which are responsible for correcting base–base mismatches and insertion/ deletion loops arising during DNA replication and recombination. This subsequently results in defective MMR that drives tumourigenesis. Although certain deletion mutations in a non-MMR gene *EPCAM* can cause Lynch Syndrome, this is because it affects transcription of *MSH2*.

Due to highly penetrant germline mutations in MMR genes, the age of onset and risk of developing CRC differs significantly between sporadic CRC and LS patients. Remarkably the risk of CRC in LS may be as high as 33–46% by the age of 70, compared to ~ 5.5% in the general population. Once a diagnosis of LS has been made, current guidance recommends affected individuals should undergo 1–2 yearly colonoscopic surveillance [2, 3]. If CRC is identified the mainstay of management is by surgical resection, either a segmental or extensive colectomy.

The diagnosis of Lynch syndrome initially relied upon clinical diagnostic criteria such as the Amsterdam 1 and 2 criteria, first published within the 1990s [4, 5]. Later emerged the Revised Bethesda Guidelines which provided a method of identifying high risk patients which should undergo tumor testing for microsatellite instability or immunohistochemistry [6]. Despite these guidelines having a sensitivity and specificity of 81 and 98% respectively, up to 28% of LS patients can be missed, with variable application of these criteria in clinical practice [7–9].

Previous suboptimal identification of LS patients has led to a recent change in guidance provided by organisations such as the National Institute for Health and Care Excellence, the Mallorca group and the Evaluation of Genomic Applications in Practice and Prevention (EGAPP) Working group who currently recommend that all patients diagnosed with colorectal cancer, irrespective of age, should be universally tested for molecular features suggestive of LS [2, 10, 11]. The aim is to improve the identification of LS patients and thus ensure patients are managed correctly, with a focus on improving patient outcomes.

Synchronous and metachronous colorectal cancers (MCC) occur more commonly in patients with LS compared to the general population [12, 13]. LS is also associated with young onset diagnosis CRC, thus there is a high risk in many patients of developing MCC after a primary tumour resection. Current American and European guidance recommend considering a more extensive resection due to retrospective case series data suggesting a greater risk of MCC after a segmental versus extended colectomy [2, 3]. Whether a segmental or total colectomy is performed remains controversial due to the lack of robust conclusive data and guidelines being based on level III evidence and grade C recommendations.

The aim of this systematic review and meta-analysis is to conclusively evaluate the risk of MCC and mortality following a segmental and extensive colectomy in LS.

## Methods

### Literature search

An extensive literature search of the PubMed database from inception (last search November 2016) was conducted. Initially the following terms were searched: “Lynch Syndrome” or “HNPCC” AND “metachronous”. This literature search was independently performed by two researchers (SS and ML).

### Eligibility criteria

The study titles were examined for potential relevance and the abstract was then reviewed. The full text was retrieved to ensure eligibility using the following inclusion and exclusion criteria.

### Inclusion criteria

1. ≥ 50 cases with previous colorectal surgical resection for colorectal cancer or cancer prophylaxis.
2. LS or HNPCC patients.
3. Median follow-up ≥ 60 months.
4. Full articles published in English within a peer reviewed journal.
5. Sufficient data was available for analyses to be conducted.

### Exclusion criteria

1. Insufficient information within the article for inclusion/exclusion to be established.
2. Study included non-colorectal cancer cases.
3. Study included data on patients without a diagnosis of LS or HNPCC.
4. Case only study.
5. Cell or animal based studies.
6. Review articles.

The bibliographies of relevant articles were also inspected for further eligible studies. In the case of any uncertainty regarding study inclusion, another investigator (KM) was consulted as a third person arbiter to assess eligibility.

### Data extraction

For eligible studies, the following data was extracted using a standardised database: title; first author; publication year; number of male and female subjects; mean age of diagnosis; mean follow up time; total number of patients in each study; total number of patients undergoing segmental; subtotal or total colectomy as well as the total number who developed MCC after each surgery and total mortality after each surgery. Extensive surgery was considered to include those patients who underwent panproctocolectomy, subtotal or Total colectomy.

If the required data was unavailable within the full text of the article or any accompanying supplementary material, this was sought through e-mail exchange with the stated correspondent(s).

### Statistical analysis

Meta-analyses were conducted to quantify the risk of MCC; risk of mortality attributed to colorectal cancer specifically and risk of mortality overall after a segmental versus an extensive colectomy. Quantitative synthesis involved calculating relative risk (RR) for three or more studies using the following formula:

$${\text{RR}}=\left( {{\text{a}}/{\text{a}}+{\text{b}}} \right)/\left( {{\text{c}}/{\text{c}}+{\text{d}}} \right)$$

For example, whereby segmental colectomy patients with (a) or without MCC (b) were compared to extensive colectomy patients with (c) or without MCC (d).

Corresponding 95% confidence intervals (CI) were deemed statistically significant if they did not cross 1. Cochrane’s Q statistic and the I^2^ test were used to determine the degree of variation between studies not attributable to chance [14, 15]. I^2^ values ranged from 0% indicating homogeneity to 100% indicating heterogeneity. If the I^2^ value was between 50 and 100%, the DerSimonian and Laird random effects method was used to generate pooled RRs [16]. If the I^2^ value was between 0 and 50% then the Mantel–Haenszel fixed effects method was used [17].

Subgroup analysis was performed to investigate if there was a significant difference in risk of MCC after segmental versus extended colectomy in patients with LS with a confirmed germline mutation, and separately assessing risk in those patients who met Amsterdam criteria alone but in whom germline status was uncertain.

Sensitivity analysis was achieved by removing each study individually and repeating the analysis. This assessed whether the results were influenced by a single study.

Publication bias was assessed using funnel plots and Egger’s test. An asymmetrical funnel plot signified the possible presence of publication bias. Publication bias was quantitatively assessed using Egger’s test, however restricted to meta-analyses containing 10 or more studies as per recommendations outlined by Sterne et al. [18, 19]. A p value of < 0.05 after Egger’s test suggested publication bias.

Statistical analyses were performed using the Metafor package in R (Version 3.3.2) [20].

## Results

**Fig. 1:**
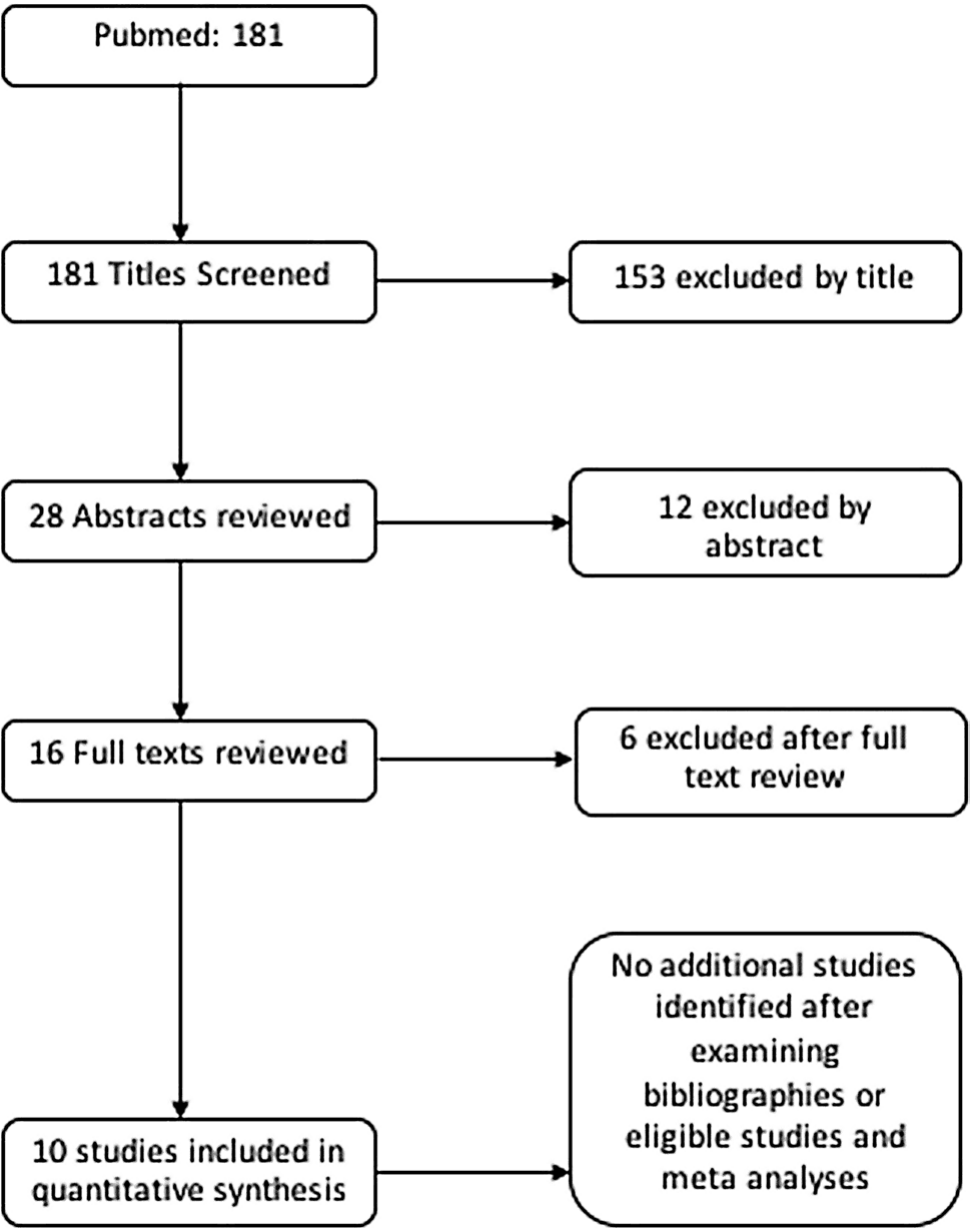
Flow chart displaying the exclusion and inclusion of studies within the meta- analysis

A search of the PubMed database yielded 181 studies. After screening titles and abstracts 165 papers were excluded and 16 studies were reviewed in full. After thorough review of these studies, ten were eligible for inclusion in quantitative synthesis (Fig. 1).

Pooled data identified 1389 patients followed up for a mean of 100.7 months with a mean age of onset of 45.52 years of age (Table 1). 1119 patients underwent segmental colectomies with risk of MCC in this group of 28.2% at the end of follow-up (Table 2). 270 patients had extensive colectomies with a MCC risk of 4.7%, and 0% in those with a panproctocolectomy (Table 1).

**Table 1:** Study characteristics

Study	Year	Germline mutation or Amsterdam only	Total cases	Gender (male %)	Mean follow-up	Mean age diagnosis	Range of age of diagnosis	Segmental Colectomy	MCC after Segmental colectomy	Extended colectomy	MCC after Extended colectomy
Kalady [34]	2012	Amsterdam	296	47.9	104	47.9	–	253	55	43	3
Van Dalen [35]	2003	Amsterdam	93	n/a	156	47	26–81	70	16	23	0
Cirillo [32]	2013	Amsterdam	65	58.5	72	47	–	54	15	11	2
Vasen [36]	1993	Amsterdam	54	n/a	69.6	45	17–88	37	8	17	2
Parry [13]	2011	Germline	382	51	108	46	–	332	74	50	0
Cappel [37]	2002	Germline	139	n/a	81.6	46	24–78	110	13	29	1
Natarajan [38]	2010	Germline	106	39.6	144	45.5	–	69	23	37	4
Kim [39]	2017	Germline	106	52.8	68.1	43.5	24–82	76	13	30	0
Win [25]	2013	Germline	88	44	132	42.8	–	79	21	9	0
Stupart [28]	2011	Germline	60	59.2	72	44	–	39	8	21	0

**Table 2:** Total number of patients and risk of MCC in segmental and extensive colectomy

	Amsterdam Criteria	Germline mutation	Overall %
Number of patients			
Segmental	414	705	1119
Extensive	94	176	270
Median follow up (months)	88.0	95.0	92.8
Risk of MCC (%)			
Segmental	22.7	21.8	22.7
Extensive	7.4	2.8	4.7

There was no significant difference in MCC risk between Amsterdam criteria and germline mutation cohorts

**Fig. 2:**
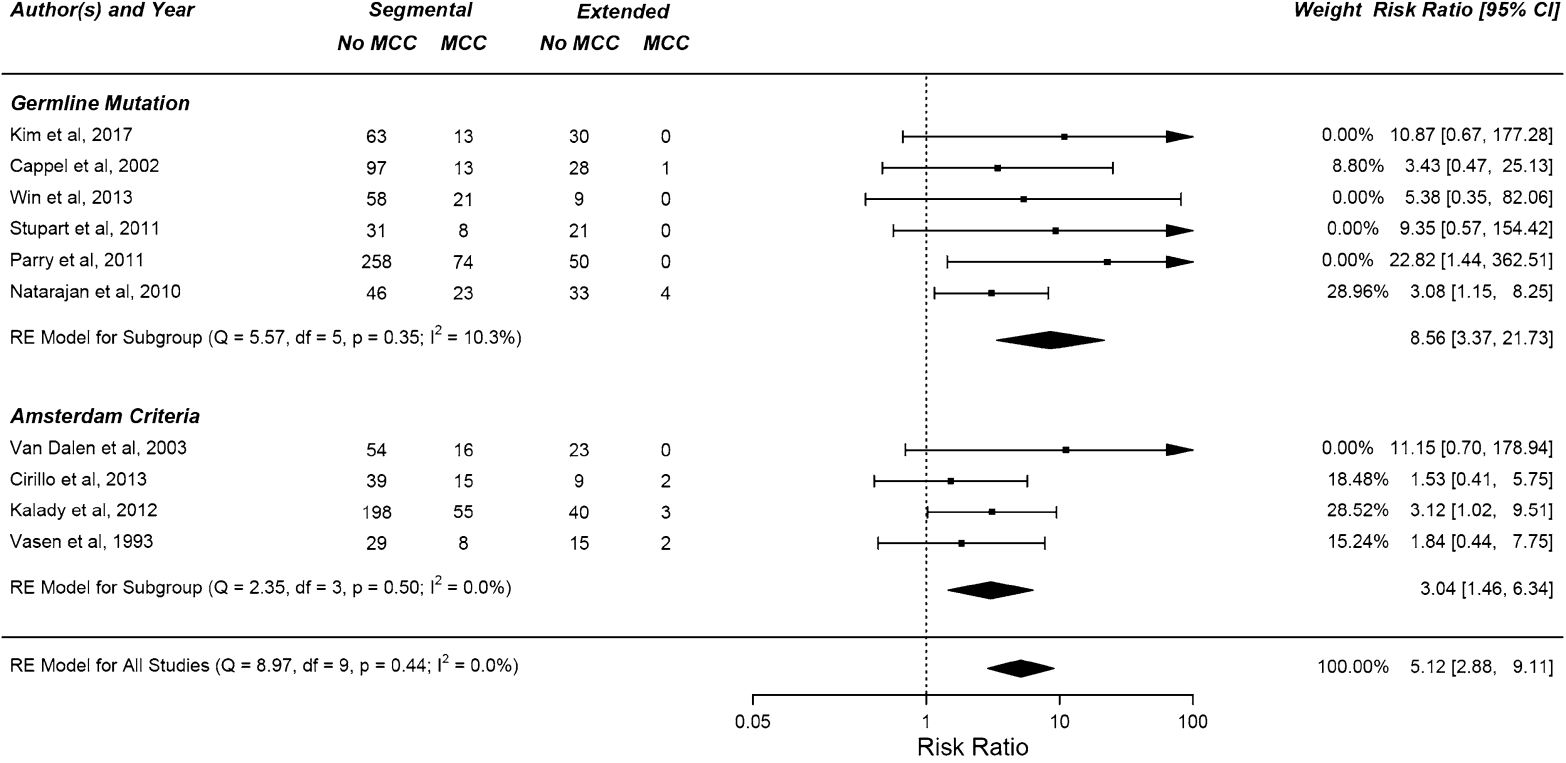
Forest plot displaying the risk of metachronous colorectal cancer after a segmental colectomy versus Extensive colectomy, with a higher relative risk amongst patients with a germline mutation

A segmental colectomy was significantly associated with an increased risk of MCC (RR = 5.12; 95% CI 2.88–9.11; Fig. 2). The relative risk of MCC after a segmental colectomy versus an extended colectomy was 8.56 (95% CI 3.37–21.73) and 3.04 (95% CI 1.46–6.34) in patients with a confirmed LS germline mutation and patients with LS diagnosis using the Amsterdam criteria respectively (Fig. 2). There was no statistically significant difference in the risk of MCC between AC and GM cohorts (p = 0.5, Chi-squared test). The relative frequencies of germline mutations in each study is presented in Table 3. Only 2 of the studies incorporated data about all 4 MMR genes, and only one specifically included data about *EPCAM* mutations.

**Table 3:** Frequency of MMR gene germline mutations in this meta-analysis

MMR gene mutated	Total	Kim	Cappel	Stupart	Parry	Win	Natarajan
MLH1	319	64	31	24	116	18	66
MSH2	273	27	32	15	104	55	40
MSH6	32	4	5	n/a	19	4	n/a
EPCAM	11	11	n/a	n/a	n/a	n/a	n/a
PMS2	21	n/a	n/a	n/a	19	2	n/a

*n/a* not available

**Fig. 3:**
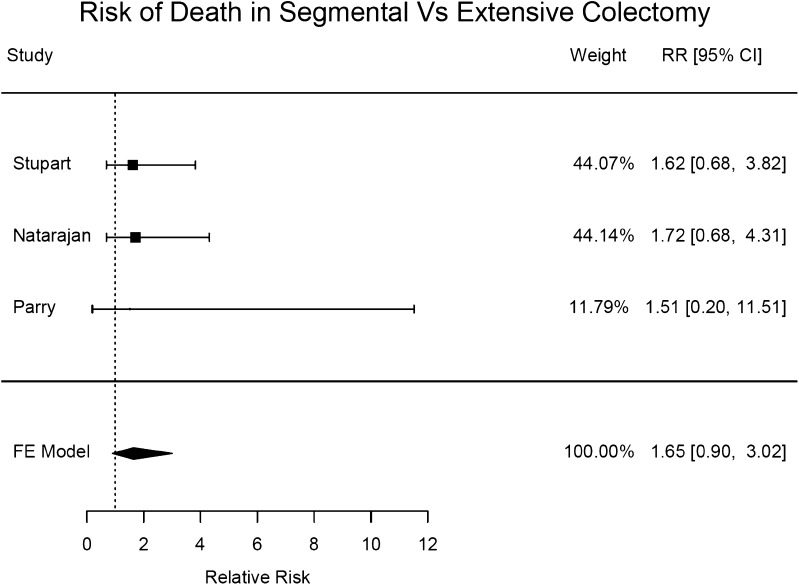
A forest plot displaying the risk of mortality after a segmental colectomy versus extensive colectomy

Although there was a trend towards higher mortality in those who underwent segmental colectomy, no significant association with mortality was identified (RR = 1.65; 95% CI 0.90–3.02; Fig. 3).

**Fig. 4:**
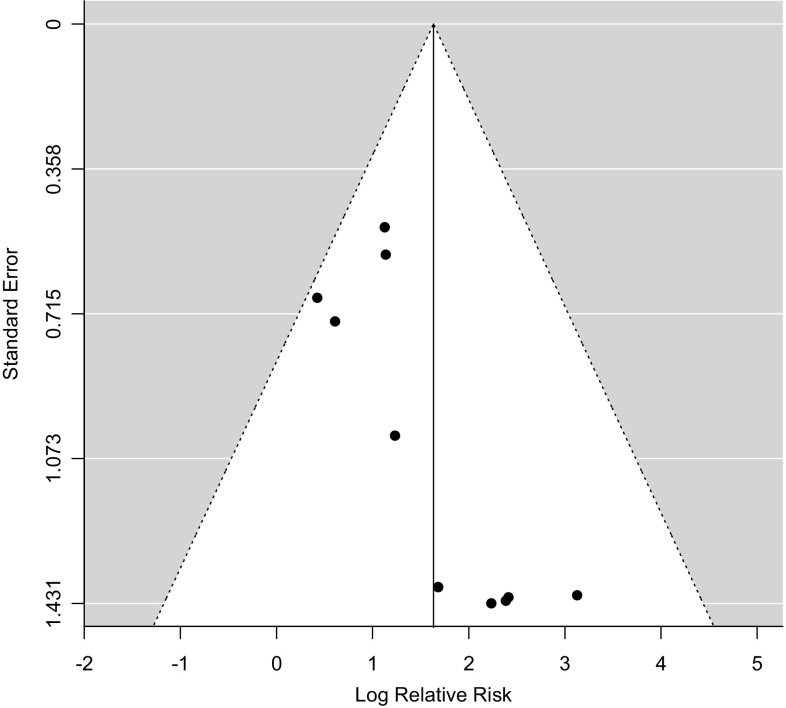
Funnel plot assessing publication bias for the risk of metachronous colorectal cancer in segmental versus extended colectomy meta-analysis

**Fig. 5:**
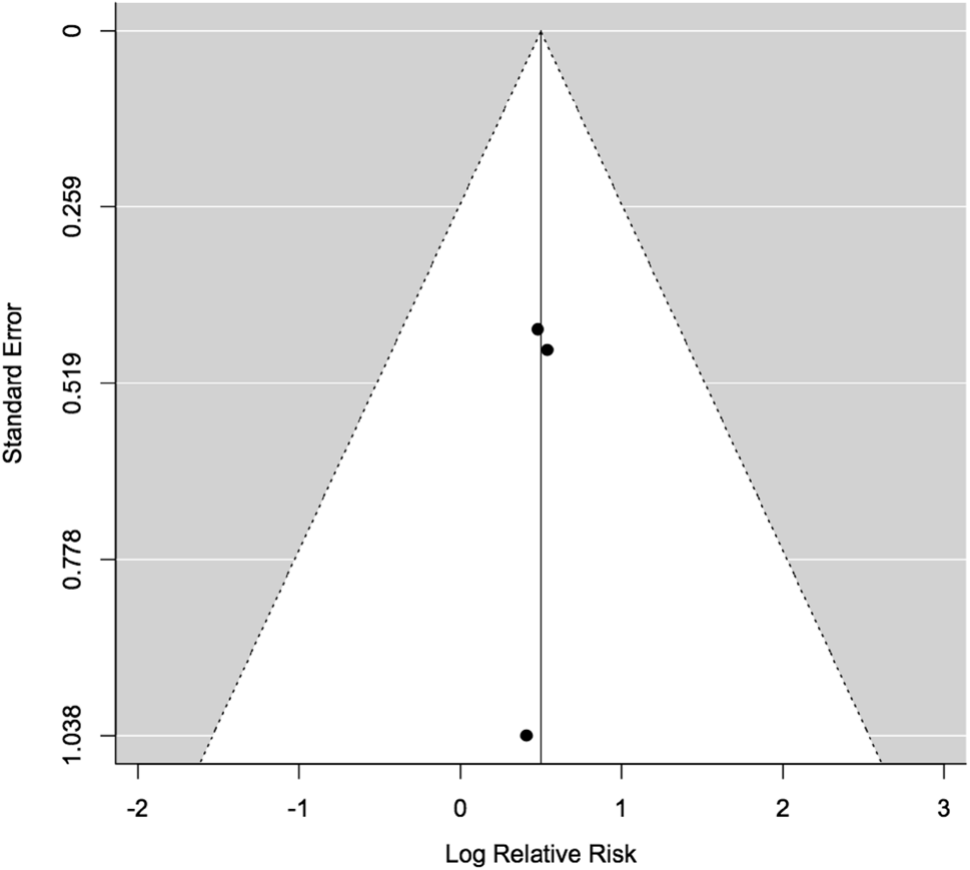
Funnel plot assessing publication bias for the risk of death in segmental versus extended colectomy meta-analysis

Funnel plots for both meta analyses were symmetrical (Figs. 4, 5). There were fewer than ten studies present within the mortality meta-analysis therefore Egger’s test was only performed for the risk of MCC meta-analysis. Egger’s test supported there was no evidence of publication bias (p = 0.0745).

## Discussion

The surgical management of Lynch Syndrome remains controversial. There is a lack of robust evidence supporting the hypothesis that there is a greater risk of MCC after a segmental colectomy versus extensive colectomy. Our study shows that there is a five times greater risk of MCC after a segmental colectomy when compared to an extensive colectomy. Although a trend towards decreased mortality in the extensive colectomy cohort is suggested by this meta-analysis, this is not statistically significant. Despite these findings an individualised approach for each patient should be utilised.

It must be recognised that patient centred care is paramount and patients should not only be counselled on the significantly greater risk of MCC after a segmental compared to an extensive colectomy, but also on the impact that more extensive surgery may have on morbidity. Steel et al. demonstrated in patients who were at high risk of MCC that they were well informed about the surgical procedure, however they feared recurrence and information relating to surgical outcome, recovery and lifestyle adjustments were inadequate [21].

Extensive surgery includes a pan-proctocolectomy or a subtotal colectomy. Patients undergoing a pan-proctocolectomy should be counselled that this will necessitate a permanent end ileostomy or ileal anal pouch anastomosis (IPAA) which will require lifestyle and dietary adaptation and has been reported to negatively impact up to 50% of patient’s sex lives [22]. Alternative extensive surgery includes a total colectomy whereby an ileorectal anastomosis is formed or a proctectomy can be performed at later date and an IPAA created. A pan-proctocolectomy will eliminate the risk of MCC however with a total colectomy the risk of a rectal cancer remains. Although proximal cancers are a hallmark of LS, it has been found that up to 15% of tumours can occur within the rectum [23]. In addition it must be acknowledged that there is greater morbidity following an extended colectomy with an increase in bowel motions, poorer functional outcome and reported lower quality of life [24].

A pan-proctocolectomy would be the optimal choice to eliminate a patient’s risk of MCC, especially if the primary tumour has occurred within the rectum. If a patient opts against this and prefers a total colectomy and ileorectal anastomosis or IPAA, then regular endoscopic surveillance of the remaining rectum would be required due to the risk of a rectal cancer. Where a segmental colectomy is the surgical management of choice regular endoscopic surveillance would be sensible due to the five times greater risk of MCC in such patients.

Despite there being a significantly increased risk of MCC, the choice of surgical management has no significant effect on mortality. However, it can logically be assumed that a cancer recurrence, need for repeat surgery and anaesthesia would all increase one’s risk of mortality. Despite our findings contradicting this, the limitations of the mortality meta-analysis must be recognised. There were only 3 studies (548 patients) that included mortality data. Before definitive conclusions are made, the meta-analysis should be repeated when more studies with mortality data becomes available. We therefore suggest that further studies investigating the effect of the choice of surgery on mortality should be conducted and authors should make this data available to allow for a conclusive meta-analysis to be conducted.

It appears that patients with higher penetrance forms of LS may be at the greatest risk of MCC. Germline specific data reveal that most of the cases in the meta-analysis were patients with *MLH1* and *MSH2* mutations, with only two of the studies reporting mutations for *MSH6* and *PMS2* mutations as well. Thus the high risk of MCC in the segmental colectomy cohort is largely due to patients with *MLH1* and *MSH2* mutations, and therefore germline specific information must be taken in to account when counselling patients about the risks of surgery [13, 25].

The choice of surgical management should also take into account the patient’s age and co-morbidities. Parry et al. showed that there was a cumulative increase in the risk of MCC as time following segmental colectomy increases [13]. The risk was demonstrated as 16, 41 and 62% at 10, 20 and 30 years respectively. This cumulative increase in risk should be considered along with the impact upon ones quality of life, particularly in a younger patient compared to a more elderly patient. Elderly patients may also have more co-morbidities further increasing their risk of mortality and morbidity following more extensive surgical intervention. Lynch syndrome is also associatd with extra colonic malignancies such as endometrial and ovarian malignancy. The presence of a concomitant extra colonic malignancy should be taken into consideration when selecting a segmental or extended colectomy.

A systematic review and meta-analysis to ascertain the risk of MCC following a segmental colectomy compared to extensive colectomy has been performed by Heneghan et al. [26] and Anele et al. [27]. There are key differences in the statistical analysis and methodology which makes the current study more advantageous.

Firstly, it is not possible to reproduce the odds ratios calculated for 5 out of 6 of the individual studies within the Heneghan study. 3 out of the 6 studies included in the meta-analysis by Heneghan et al. have also been included by Anele et al. and again the odds ratios calculated for these studies do not correlate. This suggests that they’re may have been an error in statistical analysis of the data by Heneghan at al, thus producing an unreliable overall odds ratio for the risk of MCC. Furthermore Anele and colleagues have made an error when extracting data from the study by Stupart et al. [28]. Mortality following metastatic colorectal cancer following an extensive colectomy has been extracted as opposed to the number of patients with MCC after an extensive colectomy.

Anele and colleagues argue that as the Heneghan study includes patients which meet the Amsterdam criteria but do not necessarily have a confirmed germline mutation, this patient demographic could include patients with familial colorectal cancer type X, which has a lower rate of MCC, thus producing bias [27]. Anele et al. try and overcome this bias by only including patients with a confirmed LS germline mutation. Contrary to this, they included patients which demonstrated microsatellite instability and/or mismatch repair deficiency from a study by Aronson et al., however only 85.4% of these patients have a confirmed germline mutation [29].

To investigate if there is a significant difference in risk between patients which meet the Amsterdam criteria (but have not undergone genetic testing) and those with a confirmed germline mutation we performed a subgroup analysis. The relative risk of MCC in these groups was 3.04 (95% CI 1.46–6.34) and 8.56 (95% CI 3.37–21.73) respectively. Despite the risk being lower within those who meet the Amsterdam criteria, there is no significant difference between the two groups.

The CAPP2 study demonstrated that regular high dose aspirin could lower the risk of developing colorectal cancer in those with LS [30]. One may argue that chemoprevention using aspirin may remove the need for a prophylactic extensive colectomy to prevent MCC. However, despite further studies required to ascertain the optimal dose of aspirin for chemoprevention, the CAPP2 study and other data (Rothwell et al.) demonstrated that the effect of aspirin is delayed and it isn’t until after a latent period of approximately a decade that the risk is significantly lowered when compared to placebo [31]. This delayed effect after 120 months occurs after the mean follow up, 100.73 months, of the patients within this study. By the point of significant effect of aspirin, the risk of MCC after a segmental colectomy compared to extensive colectomy is already increased fivefold, therefore necessitating the need for a prophylactic colectomy to lower one’s risk of MCC.

In addition to chemoprevention, the role of endoscopy in risk modification is not well established. Current international guidance recommends 1–2 yearly colonoscopic surveillance of LS carriers in order to ensure colorectal cancers are identified and managed promptly [2, 3]. Several studies have now demonstrated no significant reduction in risk of MCC in segmental and extensive colectomy patients undergoing regular biennial colonoscopic surveillance [13, 25, 32]. Perhaps more regular or higher quality colonoscopic surveillance may be more beneficial, however the efficacy and cost effectiveness of this would need to be evaluated.

## Conclusion

We conclude that there is a significantly greater risk of MCC following a segmental colectomy compared to extensive colectomy in LS patients. An individualised approach must be utilised taking into account the age, co-morbidities and genotype of the patient as well as the morbidity and impact on the quality of life the choice of surgical management may have. Patients must be counselled on the findings of this meta-analysis prior to any surgical intervention and informed, using the best available germline-specific information. Despite this study demonstrating no significant effect on mortality, better powered studies are required to investigate this further, and an existing international collaboration may be well placed to answer this and other questions [33]. The current role of aspirin chemoprevention and regular endoscopic surveillance to reduce risk of MCC over the follow-up of this analysis is not well established. However, a prophylactic extensive colectomy may be the most effective current MCC risk reduction strategy especially given the delayed effect of aspirin chemoprevention and limited effectiveness of endoscopic surveillance.
